# Methodological issues in economic evaluations of emergency transport systems in low-income and middle-income countries

**DOI:** 10.1136/bmjgh-2020-004723

**Published:** 2021-03-18

**Authors:** Richard Lilford, Dmitri Nepogodiev, Peter J Chilton, Samuel I Watson, Darius Erlangga, Peter Diggle, Alan J Girling, Mark Sculpher

**Affiliations:** 1 Institute of Applied Health Research, University of Birmingham, Birmingham, UK; 2 Department of Global Health & Development, London School of Hygiene and Tropical Medicine, London, UK; 3 Lancaster Medical School, Lancaster University, Lancaster, UK; 4 Centre for Health Economics, University of York, York, UK

**Keywords:** health economics

## Abstract

A recent systematic review identified few papers on the economic evaluation of systems for emergency transport of acutely ill or injured patients. In addition, we found no articles dealing with the methodological challenges posed by such studies in low-income or middle-income countries. We therefore carried out an analysis of issues that are of particular salience to this important topic. This is an intellectual study in which we develop models, identify their limitations, suggest potential extensions to the models and discuss priorities for empirical studies to populate models. First, we develop a general model to calculate changes in survival contingent on the reduced time to treatment that an emergency transport system is designed to achieve. Second, we develop a model to estimate transfer times over an area that will be served by a proposed transfer system. Third, we discuss difficulties in obtaining parameters with which to populate the models. Fourth, we discuss costs, both direct and indirect, of an emergency transfer service. Fifth, we discuss the issue that outcomes other than survival should be considered and that the effects of a service are a weighted sum over all the conditions and severities for which the service caters. Lastly, based on the above work, we identify priorities for research. To our knowledge, this is the first study to identify and frame issues in the health economics of acute transfer systems and to develop models to calculate survival rates from basic parameters, such as time delay/survival relationships, that vary by intervention type and context.

**Summary box**

- Despite the importance of the topic, there is very little literature available on economic evaluation of emergency transport in healthcare in low-income and middle-income countries, and none dealing with the methodological issues that this topic throws up.
- We develop a basic model for a decision maker to calculate cost per life saved for a particular condition from the correlation between survival and treatment delay, and the conditions affecting delay in a particular locality.
- We discuss how this model may be extended to deal with all emergencies, outcomes apart from survival and provision of prehospital care.
- We identify priorities for empirical studies to populate models.

## Introduction

Decisions about implementing new interventions need to reflect resource constraints; evidence-informed decisions balance the benefits of a potential intervention against the benefits that could be achieved in other ways with the same resources. Economic evaluation studies inform these decisions and generic guidelines have been drawn up to guide health economic evaluations.^1 2^ Each type of evaluation creates particular challenges and a literature has developed to inform decision models for interventions covering surgery,^3–6^ treatments that not everyone will choose even when they are freely available,^7 8^ and service/policy interventions,^9–15^ for example. We have been working on health economic models to inform investment decisions concerning emergency transport systems, such as motorised ambulance systems, in low/middle-income countries (LMICs). During the course of this work, we encountered a number of methodological issues, and turned to the literature for help. We have updated a systematic review published in 2017,^16^ but found only 12 articles concerned with the economic evaluation of emergency transport in LMICs, none of which dealt with methodological issues (Erlannga, Lilford. *Article in preparation*. 2021). Our aim in this article is to describe issues in health economic assessment of emergency transport systems and discuss potential solutions.

## Estimating effectiveness

### Direct estimates from comparative studies

Estimates of intervention effectiveness are often obtained by means of studies with contemporaneous controls. In policy and service delivery research, these studies often take the form of cluster studies. Ideally such studies would be randomised and would include baseline observations.^17^ However, our systematic review (cited above) identified no controlled studies of the effectiveness of emergency transport systems in LMICs. Moreover, even if we had found a randomised controlled study of the effectiveness of a transport system in one place, the estimate could not be used directly in a cost-effectiveness analysis in another place. This is for two reasons. First, there is a range of service specifications: transport for all emergencies versus specific conditions (eg, road traffic incidents or maternity care); transport types (eg, motor vehicle vs motorcycle with side-car) and workforce configurations (eg, with or without paramedical support). Second, the effectiveness of a given emergency transport system depends on the variegated physical factors that affect transfer times—poor or non-existent roads in Liberia, dense jungles in Papua New Guinea, swollen rivers in Mozambique, steep slippery terrain in Nepal, dense traffic in Lagos and so on. In short, a single effectiveness measure cannot suffice across the range of the different service configurations and geographical contexts. Models are therefore required to estimate the effectiveness of a particular transport system in a particular place.

## A modelling of the effectiveness of a proposed transport system in a particular context

### Framing the model

The benefits of an emergency transport system are mediated by reducing the time delay between injury or the onset of illness and treatment. The model we propose is thus built around the relationship between time and survival. We are concerned here with delay in reaching the facility where treatment will be given. We are not concerned with any delay in receiving care once an appropriate facility has been reached (sometimes called the ‘third delay’) because this is not amenable to a transport system. However, we cannot ignore the question of whether, or when, a decision to seek care is made (the ‘first delay’). This is because an emergency transport system may influence whether care is sought at all—a kind of supply-induced demand.^18^ We will therefore ‘start the clock’ at the point where a patient or carer recognises an emergency health need. We will describe the delay between this point and arrival at the facility by the shorthand ‘transfer delay’ to signify the component delay that can be reduced by a transport system. We illustrate the model outputs with a theoretical population of 100 000 over a 1-year period. We propose a basic model in the first instance dealing with survival only and assuming no treatment ‘en route’, leaving permanent disability and paramedical services to future developments of the model. We will motivate our model through the lens of a single-presenting condition—postpartum haemorrhage—as an example of an archetypal time-critical condition. We address the problem of consolidating costs and effects across all emergency conditions for later discussion.

### The basic model

We need to calculate the death rate for this population over 1 year, absent a formal transport system. Then, we will calculate the mortality given a transport system assuming, in the first instance, that it has sufficient coverage to transport all who need the service. Lives saved are based on subtracting the latter from the former.

**Figure 1:**
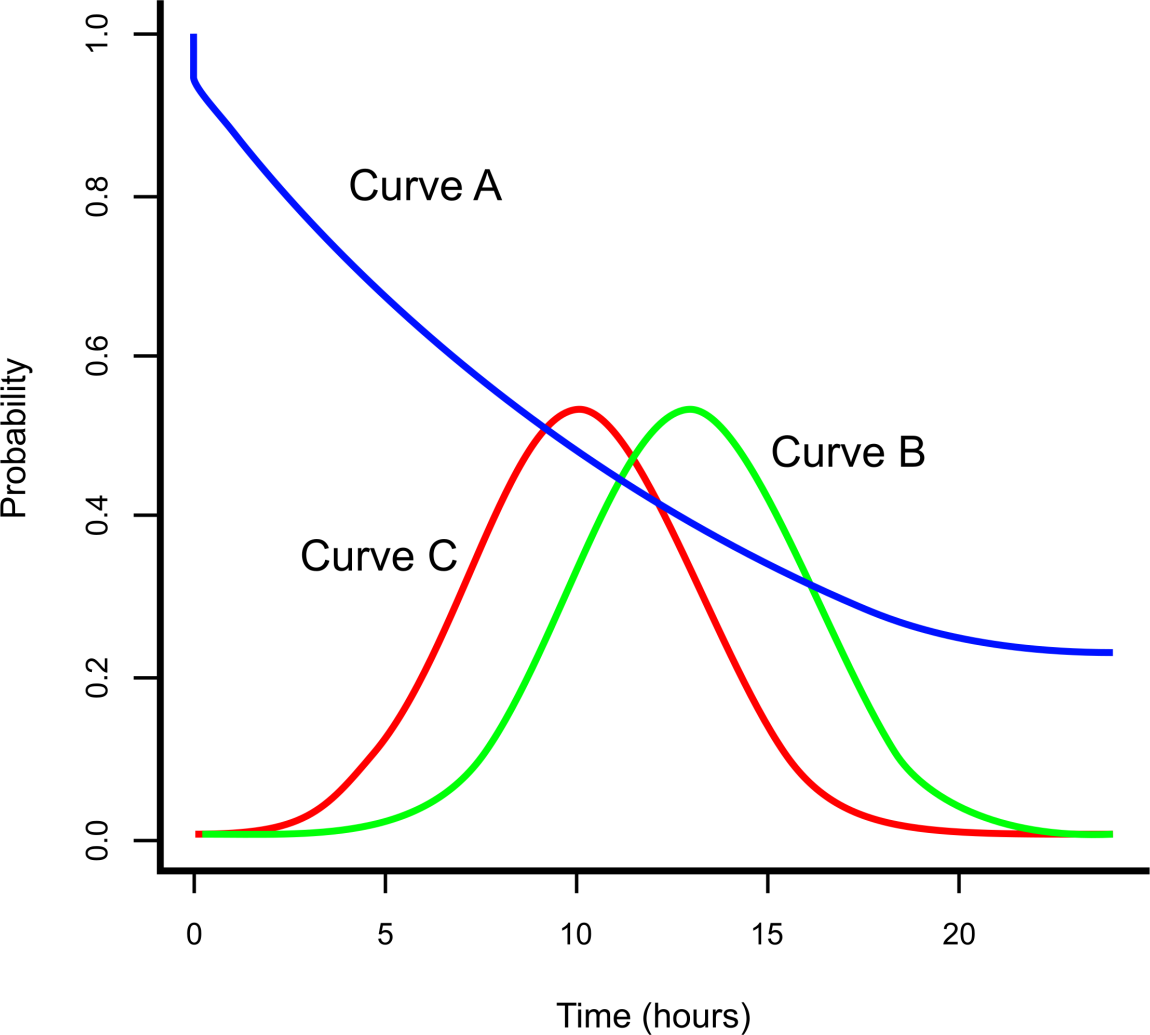
Postpartum haemorrhage. Curve A is the survival curve by time from recognition of the care need to arrival at the facility that will provide care, curve B represents the counterfactual time distribution from recognition of the need for facility care to arrival at the facility (transfer delay) under baseline conditions and curve C is the transfer delay under a proposed transport system. In order to construct curves B and C, it is necessary only to specify the shape of the distribution (assumed to be normal above), the mean transfer delays (currently and after introduction of the service) and the SD (assumed above to be the same for both curves). The uncertainty in the estimated mean would also be specified for a probabilistic analysis.

In figure 1, curve A represents the probability, *S(t),* of survival as a function of transfer delay, *t*, where *t=0* is the moment a care need is recognised. Curve A becomes ‘flat’ after a time in recognition of the fact that in most conditions some will survive even if they never reach an appropriate facility. The origin of curve A starts at a figure lower than 100% on the y axis in many (most) circumstances where some deaths are inevitable. Curve B is proportional to the probability distribution, *f(t)*, of the time taken from recognising a care need to arriving at a care facility (transfer delay) and is intended to represent a plausible scenario in some part of the world. Curve C is the counterfactual probability distribution, f*(t), under a proposed new transport system.

In online supplemental appendix 1, we develop a mathematical model to calculate the survival gain (per 100 000 of population), contingent on reducing the distribution of transfer delays from curve B to curve C.

10.1136/bmjgh-2020-004723.supp1 Supplementary data

### Modelling transfer delay

However, transfer times at baseline and the extent to which a transport service may reduce these times, will vary widely by region and country. These time estimates depend on local geographical variables, particularly population density and travel times. For example, population density is greatest in cities where people gain less in terms of transfer times than in more remote locations. Moreover, the propensity to seek care might be affected by location. Rather than specifying arbitrary probability distributions, such as curves B and C in figure 1, it would be more accurate to average over the non-uniform distribution of population density and transfer times across the area of interest, in calculating expected gains in survival.

In online supplemental appendix 2, we develop a mathematical model to calculate a distribution of transfer delays from population densities, distances and ‘transfer delays’ by location.

10.1136/bmjgh-2020-004723.supp2 Supplementary data

### Populating the basic model: parameters needed to calculate effectiveness

#### The relationship between delay and survival

A number of empirical studies concerned with the effect of delay on survival have used distance from facility as a proxy for delay. This method is flawed because such studies are subject to ‘survival’ bias: bias resulting from factors that prevent a person from being included in the denominator.^19^ The extent of the bias might be sufficient to reverse the association between distance and outcome, such that those living further away have a *lower* observed rate of death than those living nearby—a phenomenon that has been observed in both high-income^20^ and low-income^21^ settings. Distance as a proxy for survival cannot be recommended for use in decision models.

Studies that focus on the relationship between time and outcome are not without problems either. The literature is limited. In LMICs, we have found the largest literature on delay survival relationships in the context of snake bite,^22–27^ followed by obstetrics.^28^ There is a limited literature from high-income countries covering trauma^29–32^ and stroke.^33^ The literature relating to paediatric emergencies and ‘the acute abdomen’ is sparse. Moreover, as stated, it is important to be aware that introduction of a new emergency transport system may be associated with people accessing a service who previously would not have done so, creating a kind of ‘survivor bias’. This bias is unlikely to apply to major trauma or interfacility transfer, where there is likely complete enumeration of cases, but could be a significant bias in conditions such as acute childhood illness, where carers do not seek treatment in a substantial and variable proportion of cases.^34^

Where the parameter estimates are not available, very sparse or potentially biased, then expert opinion should be used^35^ in the form of probability densities.^36^ The methods to elicit these ‘Bayesian’ probability densities have been described elsewhere.^37–40^ Probabilistic sensitivity analyses can then be used to consolidate the many uncertainties mentioned above.^41^ By producing a range of estimates on the effect of reducing delay as a proxy for survival, local decision-makers can adjust the model to their context.

### Transfer delays

As stated above, two types of data are needed to calculate aggregate transfer delays under an existing and a proposed emergency transfer specification: local population densities and transfer times from different locations in an area. Evidence is available on population densities, since gridded (point) estimates of population density are available for the whole planet up to a resolution of 1 km^2^, for example, from WorldPop.^42^ Estimating transfer times is more difficult. Travel times round the globe by the fastest mode of possible transport under normal (non-emergency) conditions, can be obtained from publicly available data.^43^ However, these data do not represent transfer delays under either baseline or counterfactual circumstances. This is because transfer delays are affected by delays in mobilising vehicles, need for round trips, different speeds allowed in emergency vehicles, loading patients into vehicles and so on. And transfer delay also has to take induced supply demand into account. There is thus a great need for empirical investigation and development of exemplar models (discussed further later and in online supplemental appendix 2).

### Incidence estimates

The rates per year for various emergency conditions (R in equations (3) and (4), online supplemental appendix 1) are published for populations of 100 000 by the Global Burden of Disease studies.^44^ Modellers and users of models need to be wary of pitfalls in this step. First, definitions of conditions are applied very differently in different places. Second, and arguably more problematic, is that the definition may not fit the kind of person who is transferred. For example, the definition of postpartum haemorrhage would not necessarily correspond to the type of patient referred to hospital. In that case, the referral rates to which survival data apply may include people at higher or lower risk of survival than those to whom the survival data apply.

## Cost estimates

### Direct costs

While production costs for vehicles and the costs of staff to operate them may be reasonably stable, the costs the service must pay are influenced by a range of issues.^45^ For example, the price of an ambulance service is subject to local negotiation. The period over which fixed costs are amortised is also a local matter. Different terrain places different levels of wear and tear on vehicles. Peaks and troughs in demand must be considered, since the transport system must either tolerate clashes when not all needs can be served (thereby reducing the effectiveness of the system) or it must create redundancy (thereby driving up costs). These costs depend on the type of service contract negotiated. The notion that a given configuration of transport vehicles is or is not cost-effective is thus not tenable; just as benefits vary by context as described above, so do costs. The resources required can be described in published literature, as recommended in a recent guideline,^46^ while local decision-makers are well-placed position to translate these into costs that reflect local contexts.

### System knock-on effects

In order to estimate net costs, it is necessary to include an estimate of the extent to which hospital costs may be affected by a change in the number of people reaching hospital when a service is provided versus when it is not—supply-induced demand. Like other community interventions, an emergency transport service may move bottlenecks ‘upstream’. In the single study we located on this topic, a voucher scheme for round-trip transport to a hospital for obstetric care in Uganda resulted in a threefold increase in referral compared with a control district.^47^ More studies of this type are required, as we discuss later, but examination of verbal autopsy reports, such as the Million Deaths Study in India,^34^ can provide an indication of ‘pent-up’ demand.

## Framing the decision problem

Here we discuss two broader issues relating to the decision problem: the type of service (comprehensive or condition specific) and the issue of the broader system in which a transport system operates.

### Specific versus comprehensive services

Ambulance services may provide open access across all emergency (time critical) conditions. Alternatively, an ambulance service may be hypothecated to a particular ‘client group’: road traffic incident victims or obstetric services, for example.^48 49^ Low-income countries may implement condition-specific (hypothecated) services with a view to upgrading to comprehensive services as their economies develop. It is easier to compute the benefits and costs of an ambulance service over a specific client group than for a service designed to cater for all emergencies. When the service is comprehensive, covering all emergencies, then the costs and effects must be averaged over all the conditions for which it is deployed, weighted by patient volumes. This is an enormous and potentially open-ended task. An approach to this sort of problem is to select an ‘inframarginal’ set of presenting conditions where we expect the greatest value from provision of a service. In the context of emergency transport, the inframarginal group may be restricted to major categories of emergency condition, such as major trauma, obstetric emergency, life-threatening infection in a child and ‘the acute abdomen’. The cost-effectiveness of the service is then aggregated to a population level. If the positive effect among the inframarginal group is sufficient when covering the fixed cost of the service, the marginal costs and benefits of adding other presenting conditions when spare capacity exists can be considered. That said, the issue of supply-induced demand described above must also be considered, and this issue is likely to be greater in a system catering for all perceived emergencies than a system targeted at a specific clinical groups, such as childbirth or road traffic incidents.

### Emergency transport services in a broader systems context

We have argued that emergency transport systems have knock-on effects on hospitals. In post-conflict situations, where there are no hospitals worthy of the name, it makes no sense to fund an emergency transport system. As the secondary care system of hospitals develops, so the value of transport systems will increase. There must be a theoretical optimal development pathway that could be defined in health economic (or indeed in broader economic) terms. Such a system-wide approach to service development is advocated by the WHO Choices Guidelines.^50^ Such an approach, while perhaps optimal, does not vitiate the model proposed here; a model for reduced transfer time would be an essential part of a model that sought to optimise value for money by developing services iteratively to follow an optimised pathway. We also note, that optimal as such a system-wide model might be, decision-makers are often, or usually, presented with more specific investment opportunities.

## Discussion

### Modelling outcomes of emergency transport decisions

The systematic review we quote above shows that there is little or no methodological literature in the field of economic evaluation of emergency transport. In addition, we do not find any comparative studies of the effectiveness of emergency transport systems in LMICs. Even if such studies existed, it would still be necessary to extrapolate beyond the observed effects, given the wide variety of system specifications and contexts in which they might be used. Modelling will therefore always be required in the field of emergency transport economics, although, as we discuss below, better data are needed to populate models. A crucial insight from this study is that the effects of an emergency transport system, and therefore the benefits that might derive, turn on reducing the delay to treatment (including infinite delay for the proportion of people who otherwise would not seek care at all). Thus, ‘reduced delay’ can be thought of as a type of proxy for the intervention; first estimate the effect a proposed intervention will have on delay and then use that estimate to calculate the contingent survival effect over the population of interest (equation (9), online supplemental appendix 2 refers).

### The role of the decision-maker in framing and populating the model

The high-context dependency of factors, such as reductions in transport time, means that some of the parameters or distributions required as inputs for the models simply cannot be taken from the literature and applied without modification to a new situation. This means that local decision-makers are not just asked to use judgement in interpreting the model, but must actually contribute to parameter estimates with which to populate the model. The corollary is that, where possible, decision-makers and modellers should work together. However, health economists are a scarce resource, and models are often published in the literature with the intention that they will inform practice. Given a range of published models over a wide range of scenario types, a decision-maker in a particular place could select and adapt the scenario most similar to the local context.

### Interim solutions

Pending further research, ‘back-of-the-envelope calculations’ or simple models to shed some light on a decision may help. For example, the cost-effectiveness equation model can be rearranged in such a way as to find out whether a local intervention of known cost is likely to fall below the cost-effectiveness threshold for the country concerned. Thus, given an appropriate threshold^51^ and a net cost, the model could define how effective the intervention would have to be in order to be cost-effective in a given economy.

### Research gaps

A non-exhaustive list of research imperatives is given in table 1.

**Table 1:** Some urgent research needs to inform models

**Estimating health benefit**	
Delay and outcome	More and better data on delay outcome correlations are needed but these have proven hard to come by, save for certain specific diseases such as thrombotic stroke.
Disability/quality of life effects of delay	The literature on delay and outcome focuses on death with some notable exceptions, such as stroke, in high-income countries.^52^ Yet, the effects of delay may involve many outcomes, including many types of permanent and short-term physical and mental effects. Work is needed on prognosis for survivors to inform cohort or simulation models of future mortality, quality of life and costs.
Paramedical services	Delay in getting to hospital and delay in the start of treatment differ according to the availability of people and facilities en route and how they are used. This topic has been studied in high-income countries, but remains a contentious issue in the medical literature.^53 54^
**Reducing delay by means of transport systems**	
Current transfer times	Data are needed over a range of ‘specimen’ geographical and service configuration scenarios to describe how people somehow manage to reach hospital even when there is no formal transport system in place.
Effect on transport services on transfer times	Real-time studies of implementations of improved transport services are required across service specification and contextual variables. We need to better estimate how services change proportions who set out for a facility, the time taken to reach a decision to try to reach a facility and transfer times.
Transfer routes	The above studies should document changes in pathways to care, since delay may result when a patient is taken to an inappropriate venue.
Exemplars	Building on the above, we need worked models of changes in call-out to arrival times over a range of different types of geographical area.
**Data relating to costs**	
Implementation costs	More and better information is needed on the cost of ambulance services by types and number of vehicles, human resources and type of procurement contract. We have found that policy leads are reluctant to divulge this information which is often locally negotiated.
Operational effects	It would be useful to collect data on the costs of maintaining different types of vehicle in the field and how these costs are affected by the type of contract.
Logistics	The size of vehicle fleet needed to cope with peaks and troughs and delays when repairs and spare parts are acquired should be explored using Queue theory.
Paramedical services	Costs of paramedical services to offset against benefits above, by reason for call-out.
Knock-on effects	The effects of transport services on demand (both intended and unintended) are tractable and should be more thoroughly researched.

## Conclusion

We present some issues that should be considered by anyone attempting cost-effectiveness modelling of transport systems in LMICs. There is a shortfall in health economic studies of emergency transport systems in healthcare, and we are not aware of any methodological literature on this subject. Our model is basic but can be extended to include outcomes apart from survival, prehospital care and other considerations. We offer our analysis as an early contribution to an important, under-researched and hopefully burgeoning field of enquiry.
