# Supplementary material for: Methodological issues in economic evaluations of emergency transport systems in low-income and middle-income countries
Source: BMJ Glob Health. 2021 Mar 18;6(3):e004723. doi: 10.1136/bmjgh-2020-004723 (PMC7977070; doi:10.1136/bmjgh-2020-004723)
Supplement: Supplementary data [file bmjgh-2020-004723supp001.pdf]

Appendix 1. Model to calculate the expected improvement in survival contingent on implementing an emergency transport system.

NB. This appendix should be read with reference to Figure 1 in the main text.

The proportion of survivors among patients who seek hospital treatment is:

$$P_1 = \int_0^{\infty} S(t)\, f(t) dt \qquad [1]$$

A proportion of patients, p, may not seek hospital treatment. In some conditions, such as road traffic incidents, this proportion is very low and could be ignored, but the Million Deaths Study in India (48) shows that it is material for many emergency conditions. P_0 denotes the probability of survival for such patients, which is likely to be equal to $S(t)$ for large t. Then, the proportion of survivors among all patients is:

$$p \times P_0 + (1 - p) \times P_1 \qquad [2]$$

If cases occur at a rate R per 100,000 per year, the expected annual number of deaths in the 100,000 population we posit above is:

$$E = R \times [1 - \{p \times P_0 + (1 - p) \times P_1\}] \qquad [3]$$

In practice, each of the ingredients of equation [3] will vary among individuals according to a range of individual circumstances. Survival may depend on severity of bleeding and other factors that may vary by location, and a more complex model would deal with any such factors – for example one could specify a regression model with relevant covariates. We discuss below the particular issue that people in remote locations may be less likely than people living nearby to seek hospital care. We show how the equation may be adapted to accommodate this factor.

A transport intervention will reduce transfer delay. As a result, Curve B in Figure 1 may be shifted to represent a new distribution in transfer times, $f^*(t)$, now represented by Curve C. The transport service may also shift the proportion of people who seek hospital treatment to a new value, p^*. The expected annual number of deaths is now given by equations [1] to [3] but with $f^*(t)$ and p^* replacing $f(t)$ and p. We can then proceed as follows to calculate the difference in survival rates as follows.

In Figure 1 assume an exponential survivor function (Curve A),

$$S(t) = \exp(-\lambda t), \qquad [4]$$

where λ is the rate at which the survival probability decreases with increasing transfer delay, and a Normal distribution with mean μ and standard deviation σ, truncated at 0, for the transfer delay (Curve B),

$$f(t) \propto \frac{1}{\sqrt{2\pi\sigma^2}} \exp\left\{-\frac{1}{2}\left(\frac{t-\mu}{\sigma}\right)^2\right\}, \quad t > 0. \qquad [5]$$

Then

$$P_1 = \exp\left(-\frac{\mu^2 - c^2}{2\sigma^2}\right) \times \frac{\Phi(c/\sigma)}{\Phi(\mu/\sigma)} \qquad [6]$$

where $c = \mu - \lambda\sigma^2$ and $\Phi(\cdot)$ is the standard Normal distribution function.

As an illustration, to correspond to the curves A and B shown in Figure 1, we set $\lambda = 0.07$, $\mu = 13$ and $\sigma = 3$. For curve C, we set $\mu = 10$. Then, in the absence of the transport intervention the proportion of survivors among patients who seek hospital treatment is 0.411. With the transport intervention, this proportion increases to 0.507.

The transport service is calculated to prevent a proportion of deaths among those who seek care. In the above example, this proportion is 0.096 (roughly 10%) and the number of deaths prevented for our hypothetical sample of 100,000 is $0.096 \times R \times (1 - p) \times 100{,}000$. It is possible that the distribution of transfer delays in curves B and C are skewed to the right as some people live in remote areas. In that case a gamma distribution could be fitted, using a shape and scale parameter, rather than a mean and SD.
