# Supplementary material for: Methodological issues in economic evaluations of emergency transport systems in low-income and middle-income countries
Source: BMJ Glob Health. 2021 Mar 18;6(3):e004723. doi: 10.1136/bmjgh-2020-004723 (PMC7977070; doi:10.1136/bmjgh-2020-004723)
Supplement: Supplementary data [file bmjgh-2020-004723supp002.pdf]

**Appendix 2. A model to calculate transfer delays over a locality.**

The population lives in an area of interest, $A \subset \mathbb{R}^2$, and the population density at a location, $x \in A$, is given by $r(x)$. The probability density of transfer delay associated with location $x$ is given by $f(t;x)$ (and $f^*(t;x)$ with the emergency transport service), and the probability the service will not be called out, $p(x)$, as before. Updating Equations (1) to (3) we can define the probability density of the number of deaths per 100,000 at location $x$ as:

$$E(x) = R\big[1 - \{p(x)P_0 + \big(1 - p(x)\big)P_1(x)\}\big] \quad [7]$$

where

$$P_1(x) = \int_0^\infty f(t;x)S(t)dt \quad [8]$$

and similarly we can define $E^*(x)$ using $f^*(t;x)$ and $p^*(x)$.

The expected number of lives saved across our area A, with the ambulance service, is then

$$E = \int_{x \epsilon \mathrm{A}} [E(x) - E^*(x)]r(x)dx \quad [9]$$

As stated in the text, population density (*r(x))* is available in publicly available data. However, estimating counterfactual travel times for emergency patients $f(t;x)$, travel times given an ambulance service $f^*(t;x)$, and any change in propensity to make a call-out contingent on the existence of the transport service p(x), are more difficult. This is for two reasons. First, these transfer times are heavily dependent on local circumstances. Second, there is a lack of published data on transfer times from archetypal locations under plausible service specifications. The latter is necessary to help local decision makers estimate local transfer delays. Given such estimates, the modelling task could be simplified by discretising an area into basic urban and rural areas (e.g. urban, peri-urban, rural) and by distance from hospital and major trunk roads (e.g. 0-10 km, 10-20 km, and so forth), so the integral in Equation [9] would become a sum over the different categories.

The above model could be informed by simulation models to deal with queuing problems that might affect $f(t)$ when no ambulance is available; again discussed in the main text.



Lilford R, *et al. BMJ Global Health* 2021; 6:e004723. doi: 10.1136/bmjgh-2020-004723
